# Assessing the Toxicological Relevance of Nanomaterial Agglomerates and Aggregates Using Realistic Exposure In Vitro

**DOI:** 10.3390/nano11071793

**Published:** 2021-07-09

**Authors:** Sivakumar Murugadoss, Lode Godderis, Manosij Ghosh, Peter H. Hoet

**Affiliations:** 1 Laboratory of Toxicology, Unit of Environment and Health, Department of Public Health and Primary Care, KU Leuven, 3000 Leuven, Belgium; sivakumar.murugadoss@kuleuven.be (S.M.); manosij.ghosh@kuleuven.be (M.G.); 2 Laboratory for Occupational and Environmental Hygiene, Unit of Environment and Health, Department of Public Health and Primary Care, KU Leuven, 3000 Leuven, Belgium; lode.godderis@kuleuven.be; 3 IDEWE, External Service for Prevention and Protection at Work, Interleuvenlaan 58, 3001 Heverlee, Belgium

**Keywords:** nanotoxicology, titanium dioxide, synthetic amorphous silica, agglomerates and aggregates, realistic exposure in vitro

## Abstract

Low dose repeated exposures are considered more relevant/realistic in assessing the health risks of nanomaterials (NM), as human exposure such as in workplace occurs in low doses and in a repeated manner. Thus, in a three-week study, we assessed the biological effects (cell viability, cell proliferation, oxidative stress, pro-inflammatory response, and DNA damage) of titanium-di-oxide nanoparticle (TiO_2_ NP) agglomerates and synthetic amorphous silica (SAS) aggregates of different sizes in human bronchial epithelial (HBE), colon epithelial (Caco2), and human monocytic (THP-1) cell lines repeatedly exposed to a non-cytotoxic dose (0.76 µg/cm^2^). We noticed that neither of the two TiO_2_ NPs nor their agglomeration states induced any effects (compared to control) in any of the cell lines tested while SAS aggregates induced some significant effects only in HBE cell cultures. In a second set of experiments, HBE cell cultures were exposed repeatedly to different SAS suspensions for two weeks (first and second exposure cycle) and allowed to recover (without SAS exposure, recovery period) for a week. We observed that SAS aggregates of larger sizes (size ~2.5 µm) significantly affected the cell proliferation, IL-6, IL-8, and total glutathione at the end of both exposure cycle while their nanosized counterparts (size less than 100 nm) induced more pronounced effects only at the end of the first exposure cycle. As noticed in our previous short-term (24 h) exposure study, large aggregates of SAS did appear to be similarly potent as nano sized aggregates. This study also suggests that aggregates of SAS of size greater than 100 nm are toxicologically relevant and should be considered in risk assessment.

## 1. Introduction

Manufactured nanomaterials (NMs) are, due to their unique physico-chemical properties, used in a large variety of applications. Nowadays, at least 1800 products containing NMs, ranging from personal care products to sporting goods, are in circulation in the global market [1]. Concerns regarding the human health effects of NMs are gradually increasing due to their increased production and use [2,3,4,5].

In the real world, such as in occupational exposure settings, NMs exist as primary particles, agglomerates, aggregates, or as a mixture thereof [6,7,8]. In agglomerates, the particles are loosely bound by weak forces such as Van der Waals in a reversible manner, while in aggregates, particles are irreversibly fused together by chemical bonding such as covalent or ionic bonding [9]. The term agglomerates and aggregates (AA) is included in the definition of NMs recommended by the European Union [10]. It states that “manufactured material containing particles, in an unbound state or as an aggregate or as an agglomerate and where, for 50% or more of the particles in the number size distribution, one or more external dimensions is in the size range 1 nm–100 nm”. However, the definition was recommended solely for regulatory applications without any regard for hazard. Moreover, the relevance of AA in terms of toxicological perspectives is still largely unknown.

Titanium-di-oxide (TiO_2_) and synthetic amorphous silica (SAS) are among the most widely used NMs. Due to their unique properties, they have found applications in food, cosmetics, paints, etc. [2,11,12]. TiO_2_ NMs are well known for their tendency to agglomerate [13], while SAS NMs are known to aggregate easily during their production for industrial/commercial applications [14]. Thus, to determine the influence of agglomeration and aggregation on NM toxicity, we investigated and compared in our previous studies the acute (24 h) toxicological effects of TiO_2_ NMs in different agglomeration states [11] or SAS in different aggregation states [12] in three different cell lines. The results suggested that in most cases, large agglomerates or aggregates were not less potent compared to their smaller counterparts. This indicated that the toxicity of tested NMs was not mitigated by their agglomeration/aggregation state, and therefore AA of NMs of larger size (size greater than 100 nm) appear to be toxicologically relevant.

To date, most studies have evaluated the toxic potential of NMs after short-term exposure [15,16,17]. Recently, long-term and repeated low dose exposure studies for the hazard assessment have been set up for NMs, better mimicking the real life exposure (e.g., workers in production) that occurs (often) at low doses. Biological effects induced by NMs have also shown to be different between short-term versus (relatively) long-term exposure [18,19,20]. Xi et al., performed a 21 d (3w) exposure study using vanadium dioxide (VO_2_) nanoparticles (NPs) [19]. In his study, A549 cells were repeatedly exposed to a low dose (0.2 µg/mL) of VO_2_ NPs and the authors observed a 50% decreased proliferation during sub-culturing at the end of every week. Similarly, Chen et al. (2016) also performed a 21 d exposure study and noticed that the proliferation of Caco2 cells were reduced up to 50% when repeatedly exposed to 0.5 µg/mL of silver (Ag) NPs [20]. In both studies, an increase in cytokines and reactive oxygen species (ROS) generation were associated with decreased proliferation.

In this study, we aimed to determine how different AA suspensions influence the biological responses in cell cultures repeatedly exposed to a low dose (three week study). There is no consensus to estimate the dose for long-term exposure. We estimated 0.76 µg/cm^2^ as an appropriate dose based on OELs for TiO_2_ and SAS [21,22], which corresponds to a concentration of 2 µg/mL. This dose was also determined as non-cytotoxic in short-term experiments (data not shown).

## 2. Materials and Methods

### 2.1. Preparation of Dispersions and Size Characterization

Two TiO_2_ NPs of different sizes (17 nm and 117 nm) in different agglomeration states (small and large agglomerates) were freshly prepared during each exposure as described in [11] (p. 9) and details of methods used for size characterization in stock are provided in (p. 10). Two different suspensions of SAS in different aggregation states (indicated as DE-AGGR and AGGR) were prepared. In addition, we also studied the two identified subfractions in the AGGR suspension (SuperN and PREC) as described in [12]. All suspensions were freshly prepared as described in [12] (pp. 8–9) and details of methods used for size characterization in stock are provided in (p. 9).

### 2.2. Cell Culture

The human bronchial epithelial cell line (16HBE14o- or HBE) and the human monocytic cell line (THP-1) were kindly provided by Dr. Gruenert (University of California, San Francisco, CA, USA), and the Caucasian colon adenocarcinoma cell line (Caco2) (P.Nr: 86010202) was purchased from Sigma-Aldrich (Overijse, Belgium). HBE cells were cultured in DMEM/F12 supplemented with 5% FBS, 1% penicillin-streptomycin (P-S) (100 U/mL), 1% L-glutamine (2 mM) and 1% fungizone (2.5 g/mL) while RPMI 1640 supplemented with 10% FBS, 1% P-S (100 U/mL), 1% L-glutamine (2 mM) and 1% fungizone (2.5 g/mL) was used for THP-1. DMEM/HG supplemented with 10% FBS, 1% P-S (100 U/mL), 1% L-glutamine (2 mM), 1% fungizone (2.5 g/mL) and 1% non-essential amino acids (NEAA) was used for Caco2 cells. All cell culture supplements were purchased from Invitrogen (Merelbeke, Belgium) unless otherwise stated. Cells were cultured in T75 flasks (FALCON, Corning, NY, USA) at 37 °C in 100% humidified air containing 5% CO_2_. Fresh medium was changed every 2 or 3 d and cells were passaged every week (7 d). Cells from passage 3–6 were used for experiments.

### 2.3. In Vitro Exposure Conditions

The experimental design used in this study was adapted from [19,20]. For the first exposure cycle (seven days), HBE cells, Caco2 cells, and THP-1 cells were seeded at a density of 10,000 cells/cm^2^, 5000 cells/cm^2^, and 10,000 cells/mL, respectively in six well plates (day 0). Based on cell doubling time, the cell numbers for each cell line were adjusted to attain optimal confluency at the end of the first exposure cycle. After overnight incubation (day 1), the cells were exposed to cell culture media containing 2 μg/mL or 0.76 µg/cm^2^ of different suspensions of TiO_2_ and SAS for 48 h (day 2 and 3). On day 3 and 5, the supernatant was removed; cell cultures were rinsed with warm HBSS twice and exposed to fresh cell culture media containing 2 μg/mL or 0.76 µg/cm^2^ of NMs for 48 h. On day seven, the supernatants were collected and the cell cultures were washed and trypisinized (subculturing). The cell number and viability were determined immediately and the same number of cells (10,000 cells/cm^2^, 5000 cells/cm^2^ and 10,000 cells/mL for HBE, Caco2 and THP-1, respectively) were seeded for the second exposure cycle. The remaining cells were processed/stored for further analysis such as glutathione measurements and DNA damage. The steps were repeated for second (7–14 d) and third exposure cycle (14–21 d).

### 2.4. Cell Viability and Number Determination

During each subculture step, about 10 µL of cell suspension-trypan blue mix (1:1 ratio) was loaded into the counting chamber slides and cell viability and number was determined by the countess^TM^ automated cell counter (Invitrogen, Merelbeke, Belgium). The results are expressed relative to control.

### 2.5. Total Glutathione Measurements

Reduced glutathione (GSH) was measured using a glutathione detection kit (Enzo life sciences, Brussels, Belgium) according to the manufacturer’s protocol and the protein content was estimated using bicinchoninic acid (BCA) protein assay kit (Thermo Scientific Pierce, Merelbeke, Belgium). GSH was normalized to the total protein content and the results were expressed relative to control (untreated cells).

### 2.6. Cytokine Quantification

Interleukin (IL)-8 and IL-6 were quantified using ELISA kits (Sigma Aldrich, Overijse, Belgium). The cytokines were measured in the supernatants (collected during glutathione measurement experiments and stored at −20 °C) according to the manufacturer’s protocol and the results were expressed relative to control (untreated cells).

### 2.7. Comet Assay

An alkaline comet assay kit [(Trevigen (C.No.4250-050-K), Gaithersburg, MD, USA)] was used to quantify DNA strand breaks as a measure of DNA damage according to manufacturer’s protocol. Cells treated with methyl methane sulfonate (MMS) (Sigma-Aldrich, Overijse, Belgium) 100 µM for 1–2 h served as positive control.

### 2.8. Statistical Analysis

Two independent experiments were performed in triplicate or duplicate, and data were presented as mean ± standard deviation (SD). Using GraphPad prism 7.04 for windows, GraphPad Software, La Jolla, CA, USA, www.graphpad.com, the results were analyzed with one-way ANOVA followed by a Dunnett’s multiple comparison test to determine the significance of differences compared with control.

## 3. Results

### 3.1. Dispersion and Size Characterization

#### 3.1.1. TiO_2_ Suspensions

The results of size characterization and zeta potential of TiO_2_ suspensions were already published in [11], and are therefore provided in the Supplementary Materials. Supplementary Figure S1 shows electron microscopy (TEM) micrographs of small (SA) and large agglomerates (LA) of 17 and 117 nm sized TiO_2_ NPs and Table S1 shows the sizes of different TiO_2_ suspensions characterized by different techniques. We used a standardized TEM technique in our previous study [23], which enabled us to measure the size of several thousand agglomerates in each suspension. The TEM characterization (median feret min) indicated that the size of 17 nm sized TiO_2_ in their least agglomerated condition (indicated as 17nm-SA) was 33 nm while it was 120 nm in their strongly agglomerated condition (17nm-LA). The sizes of small (117nm-SA) and large agglomerates (117nm-LA) of 117 nm sized TiO_2_ were 148 and 309 nm, respectively, indicating that there were also clear differences in sizes between SA and LA of both TiO_2_ NPs. Although differences between SA and LA were observed in the sizes measured by dynamic light scattering (DLS) and particle tracking analysis (PTA), technical issues involved in observing larger sizes were discussed in [11] (p. 8). To verify the stability of agglomerates, TiO_2_ stock suspensions were diluted to 100 µg/mL in complete culture medium (CCM) and sizes were measured using DLS at 0 h and 24 h (Supplementary Table S2). The sizes of all agglomerates remained similar at 0 and 24 h, indicating their good stability over time.

#### 3.1.2. SAS Suspensions

The results of size characterization and zeta potential of SAS suspensions were already published in [12], and are therefore provided in the Supplementary Materials. Figure S2 shows the bright field (BF) microscopic image of different SAS suspensions and Table S3 shows the sizes of different SAS suspensions characterized by different techniques. SAS is a material with aggregates of broad size range (few hundred nm to few tenths µm). Thus we used different techniques (such as sonication and vortexing) to obtain suspensions with different sizes. The TEM characterization of sonicated suspension (de-aggregated, indicated as DE-AGGR) was quite straightforward and their mean feret min size was determined as 28 nm. However, using TEM and DLS, we were not able to determine the difference in sizes of other suspensions such as a vortexed suspension (aggregated, AGGR) or a suspension fractionated from AGGR [non-precipitating fraction (SuperN) and precipitating fraction (PREC)]. Thus, we used bright field microscopy and sizes of SuperN and PREC aggregates were roughly determined as 2.5 and 25 µm, respectively. By combining different techniques, we were able identify the differences in sizes between these SAS suspensions. To verify the stability of aggregates, SAS stock suspensions were diluted to 100 µg/mL in CCM and sizes were measured using DLS at 0 and 24 h. Despite knowing that AGGR and PREC sizes were not reflecting the realistic size distribution due to their quick sedimentation while performing DLS measurements, we provided the results in Supplementary Table S4. Thus, we only consider the sizes of DE-AGGR and SuperN aggregates. The sizes of DE-AGGR remained similar at 0 and 24 h, while the size of SuperN aggregates slightly reduced after 24 h.

### 3.2. Comparison of Biological Responses

#### 3.2.1. TiO_2_ Suspensions

**Figure 1:**
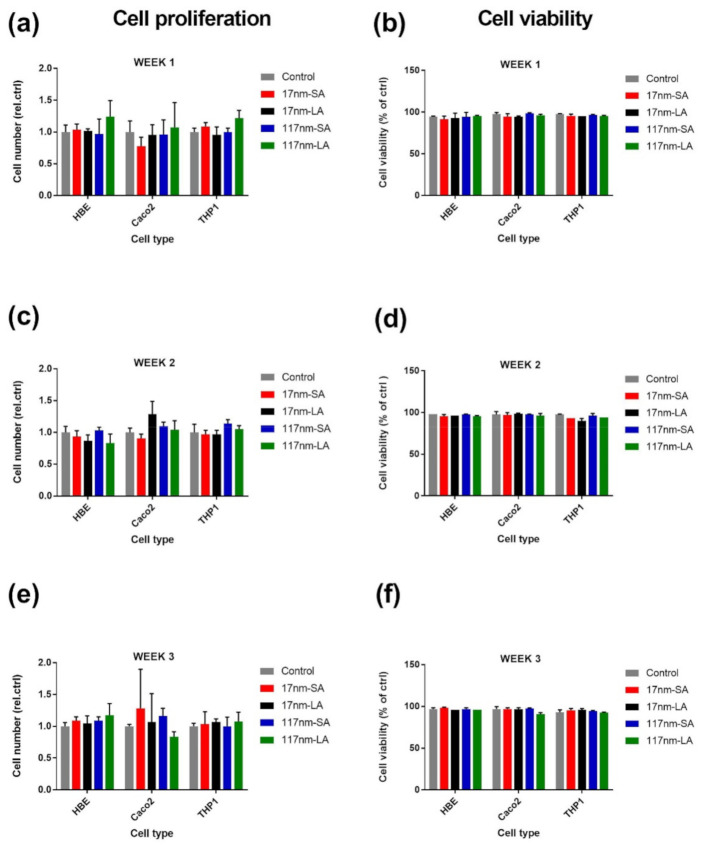
Effect of repeated low dose exposure to TiO_2_ suspensions on cell proliferation and viability. Cell proliferation profiles (**a**,**c**,**e**) and cell viability (**b**,**d**,**f**) was measured in different cell cultures after first (**a**,**b**), second (**c**,**d**), and third exposure cycle (**e**,**f**). Data are expressed as means ±SD from two independent experiments performed in duplicates. SA—small agglomerates; LA—large agglomerates.

**Figure 2:**
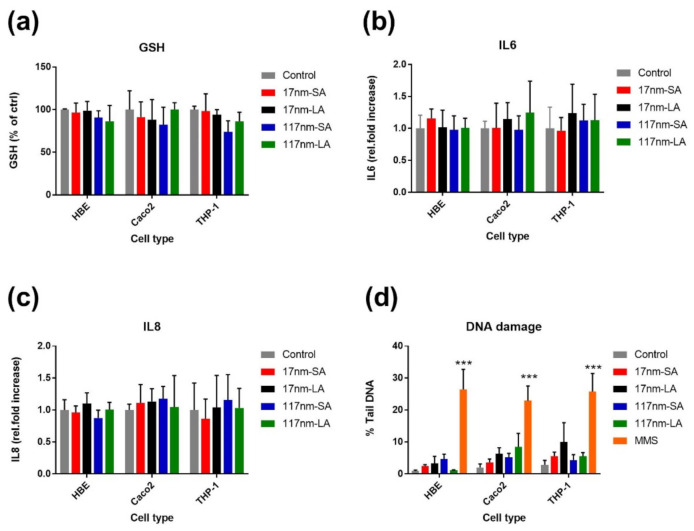
Effect of repeated exposure to TiO_2_ suspensions (0.76 µ/cm^2^) on biological responses. Total glutathione (GSH) (**a**), IL-6 (**b**), IL-8 (**c**), and DNA damage (**d**) was measured in different cell cultures after third exposure cycle. Data are expressed as means ±SD from two independent experiments performed in duplicate. *p* < 0.001 (***) represents significant differences compared to control (One-way ANOVA followed by Dunnett’s multiple comparison test). SA—small agglomerates; LA—large agglomerates.

The proliferation profiles and viability of cell cultures determined at the end of every week in three different cell lines is shown in Figure 1. None of the TiO_2_ suspensions did affect the cell proliferation and viability at the end of any exposure cycles. Compared to control, no significant effects for any of these suspensions were noticed for glutathione depletion, IL-8 and IL-6 increase, or DNA damage (Figure 2), which were evaluated after the third exposure cycle only.

#### 3.2.2. SAS Suspensions

**Figure 3:**
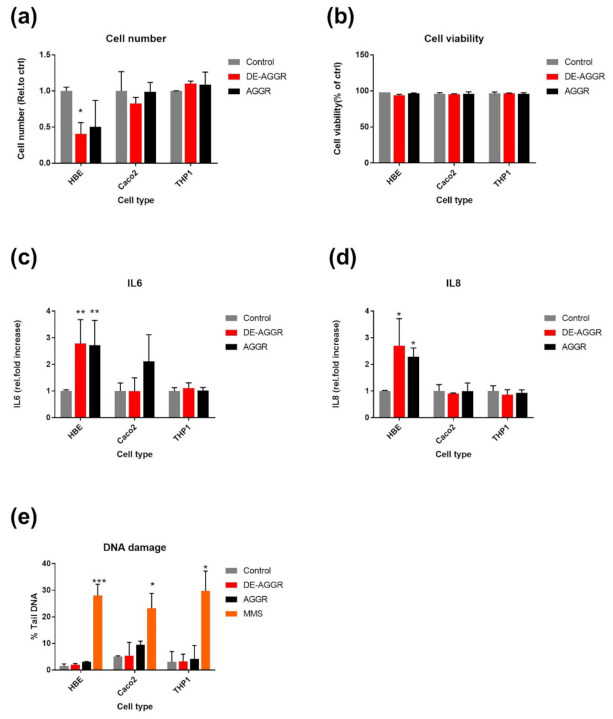
Effect of repeated exposure to SAS suspensions (0.76 µ/cm^2^) on biological responses. Cell proliferation (**a**), viability (**b**), IL-6 (**c**), IL-8 (**d**), and DNA damage (**e**) was measured in different cell cultures after third exposure cycle. Data are expressed as means ± SD from two independent experiments performed in duplicate. *p* < 0.05 (*), *p* < 0.01 (**) and *p* < 0.001 (***) represent significant differences compared to control (One-way ANOVA followed by Dunnett’s multiple comparison test). DE-AGGR—de-aggregated suspension; AGGR—aggregated suspension.

Figure 3 shows the summary of biological responses evaluated in cell cultures exposed to SAS after the third exposure cycle. DE-AGGR reduced HBE cell number significantly compared to control but AGGR did not. DE-AGGR and AGGR induced a significant increase in IL-8 and IL-6 only in HBE cell cultures. As observed for TiO_2_, SAS did not induce significant DNA damage at the tested dose. Importantly, no significant effects were noticed in the Caco2 or THP-1 cell lines in any of the biological endpoints measured. These preliminary results suggest that SAS induces biological responses at the tested dose, and it would be interesting to study and compare all fractions of the AGGR suspensions of SAS. In a set of follow-up experiments, we used only HBE cells to investigate other SAS suspensions for their effect on cell number, viability, GSH, IL-6, and IL-8. We planned two exposure cycles (two weeks) with a view to the potential recovery after discontinuing exposure, the third observation week was a recovery period without SAS exposure.

**Figure 4:**
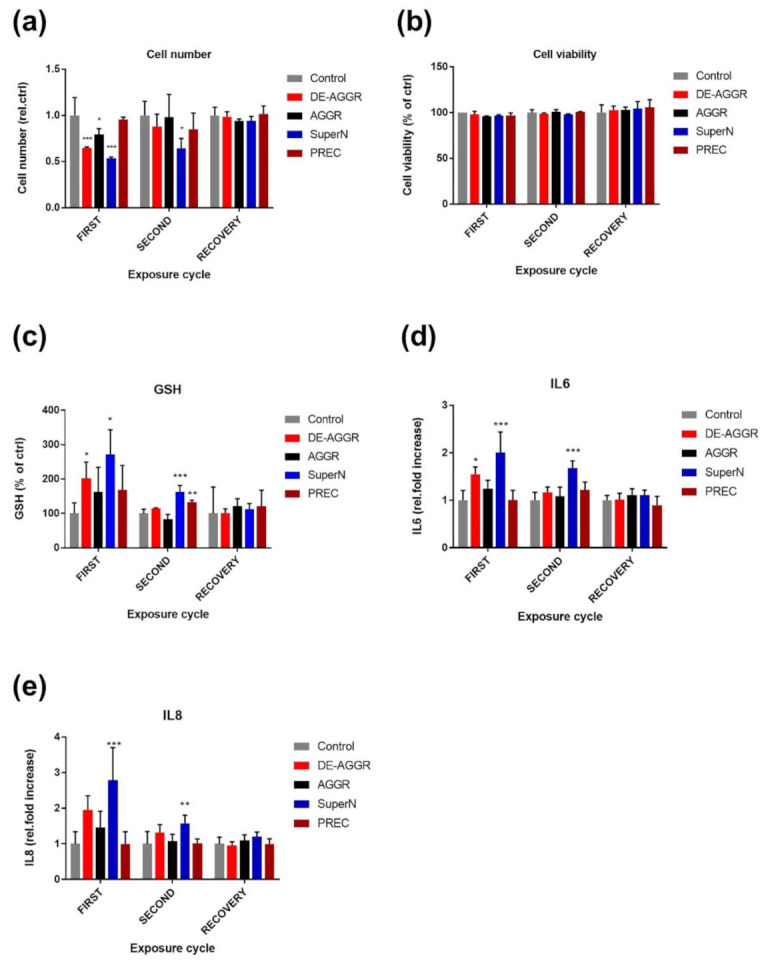
Effect of repeated exposure to different SAS suspensions (0.76 µ/cm^2^) on biological responses. Cell proliferation (**a**), cell viability (**b**), total glutathione levels (GSH) (**c**), IL6 (**d**), and IL8 (**e**) were measured in HBE cell cultures after different exposure cycles. Recovery denotes a week of exposure to cell culture medium without SAS. Data are expressed as means ±SD from two independent experiments performed in duplicate. *p* < 0.05 (*), *p* < 0.01 (**) and *p* < 0.001 (***) represent significant differences compared to control (One-way ANOVA followed by Dunnett’s multiple comparison test). DE-AGGR—de-aggregated suspension; AGGR—aggregated suspension; SuperN—non-precipitating suspension; PREC—precipitating suspension.

Effect on proliferation and viability: To determine the effect on cell proliferation, we measured cell number and cell viability at the end of each exposure cycle and recovery period (Figure 4). DE-AGGR and SuperN fractions strongly affected the cell growth at the end of the first exposure cycle. Compared to untreated cells, the DE-AGGR and SuperN fractions decreased the cell growth to about 65 and 50%, respectively (Figure 4a). AGGR, on the other hand, inhibited cell growth by about 20%. Surprisingly, DE-AGGR and AGGR exposed cell cultures recovered and remained similar compared to controls at the end of the second exposure cycle, but SuperN exposed cell cultures still exhibited decreased cell growth (about 35%). Despite a mild and non-significant decreasing trend observed at the end of the second exposure cycle, PREC fractions did not affect the cell growth significantly after both exposure cycles. After a week of recovery, all cell cultures exhibited similar growth to control. Compared to untreated controls, none of these suspensions affected the cell viability significantly after exposure cycles and recovery cycle (Figure 4b).

Effect on total glutathione: At the end of the first exposure cycle, we observed that the GSH levels had increased to about 200 (±47) and 270 (±71) % in DE-AGGR and SuperN exposed cells, respectively, compared to untreated cells (Figure 4c). Additionally, an upward trend was noticed for AGGR and PREC fractions but was not significant. The GSH levels in DE-AGGR exposed cell cultures returned to normal after the second exposure cycle while the GSH levels were still high in SuperN exposed cells (about 160 ± 18%). Interestingly, cell cultures exposed to PREC also showed mild but significantly increased GSH levels (about 130 ± 5%). The GSH levels in all the exposed cell cultures returned to normal after seven days of recovery period.

Effect on cytokine secretion: After each cycle, cytokines such as IL-6 and IL-8 were quantified in the supernatant of cell cultures (Figure 4d,e, respectively). SuperN fractions resulted in a nearly 2-fold increase in IL6 and IL8 after one week exposure and remained significantly increased at the end of second week. Like at other endpoints, DE-AGGR fractions induced a significant increase only at the end of the first week of exposure. Compared to controls, AGGR and PREC did not affect the levels of IL-6 and IL-8. After a week of recovery, no differences between suspensions were found.

## 4. Discussion

In this study, we aimed to determine how different AA suspensions influence the biological responses in cell cultures repeatedly exposed (3w study) to a dose of 0.76 µg/cm^2^. Neither of the TiO_2_ dispersions induced significant effects, while SAS suspensions generated by sonication (DE-AGGR) induced some effects compared to control and vortexed suspensions (AGGR), mainly in HBE cells. In an additional study comparing two weeks’ exposure of HBE cells with four different SAS suspensions (AGGR, DE-AGGR, SuperN or PREC), it appears that SuperN did not appear to be less potent compared to De-AGGR, which is in line with the acute effects (24 h) described in our previous study [12].

In our recent study [11], we showed that TiO_2_ agglomeration influences the toxicity/biological responses in high dose short-term exposure (24 h), while in this repeated low dose study, neither TiO_2_ exposure nor their agglomeration influences the biological responses. In a three week exposure experiment, no cytotoxic effects were observed in human mesenchymal stem cells although nano-TiO_2_ was detected in the cytoplasm [24]. Kocbek et al. (2010) did not notice any significant effects in keratinocytes repeatedly exposed to 10 µg/mL of TiO_2_ NPs for three months, while at the same concentration ZnO NPs induced a decrease in mitochondrial activity, abnormal cell morphology, and disturbances in cell-cycle [25]. Vales et al. (2014) suggested that BEAS2B cells repeatedly exposed to 20 µg/mL for four weeks showed potential for carcinogenicity (soft agar assay) [26]. These results suggest that the TiO_2_ dose used in our experiments (2 µg/mL) might not be sufficient to induce adverse effects. We based the choice of 2 µg/mL on our earlier ‘acute’ exposure experiments without cyto/genotoxicity, which now appears to be a relatively safe dose after three weeks of exposure.

In this study, DE-AGGR, the least aggregated and nano-sized SAS, induced a more pronounced effect than AGGR at the same mass concentrations. Our characterization revealed that 75% of total mass of AGGR was composed of PREC aggregates, which is about 25 µm in size [12]. PREC aggregates, when studied separately, did not induce any effects. Given their larger size, such aggregates are less likely to be taken up by the cells, and therefore induced no effects. This indicates that overall biological activity of SAS NMs in their manufactured form was reduced due to aggregation.

Similar to acute studies, SuperN fractions of AGGR suspension exhibited noticeable biological activity in a low dose repeated exposure study. The most quoted nanotoxicity paradigm is “the smaller the size of the NPs the greater the toxicity/biological responses”. Likewise, several short-term cytotoxicity studies showed that nano-sized particles are more biologically active than micron-sized studies [27,28,29]. In a recent study, bronchial cells repeatedly exposed to a low dose of VO_2_ NPs for three weeks showed greater adverse response for nano-sized particles than micron-sized particles [19]. In contrast to these observations, we observed that SuperN aggregates of size about 2.5 µm showed similar biological activity to nano-sized fractions. This suggests that larger aggregates of NP may not necessarily be considered biologically less active and highlights the need for a case-by-case analysis.

The size of SuperN aggregates (2.5 µm) is far greater than DE-AGGR aggregates (100 nm) yet falls under the category of respirable particles [30]. Therefore, exposure and hazard assessment of such fractions is valuable since commercially available SAS can be composed of small and large aggregates. We also observed that PREC was the least biologically active. Considering the size of the aggregates play a key role in determining its toxicological relevance, these findings could also contribute to the “safe-by-design” of SA, by considering aggregation as a critical factor.

Studies have indicated that the effects induced by NMs were different for short-term and long-term exposure [18,19,20]. In this study, we noticed an increase in glutathione levels after the first exposure cycle (one week) for both DE-AGGR and SuperN. Therefore, in addition to a three week exposure study, we also investigated the in vitro effects after short-term high dose exposure to SAS exposure under the same experimental conditions (Supplementary Figure S3). In short-term exposure (24 h), mild cytotoxicity (Figure S3b) and total glutathione depletion (Figure S3c) was observed at high concentrations of DE-AGGR and SuperN. Glutathione depletes when excessive ROS is produced. Several short-term studies have shown that SAS reduced glutathione levels [31,32,33,34], which is in agreement with our findings. This indicates that glutathione depletion is an earlier effect of short-term cytotoxicity while increased glutathione production is possibly a sign of a protective effect to prevent further damage. Further, decreased cell proliferation in a three week study is also consistent with an increase in IL-8 and IL-6, while only IL-8 was consistent with short-term cytotoxicity (Figure S3e). These results indicate that cell cultures may respond to NM differently depending on the modes of exposure (short-term high dose or low dose repeated exposure).

To have a view on the potential role of survival cells from first cycle exposure, the cells from the first cycle exposure were passaged and repeatedly exposed in the second cycle. At the end of second exposure cycle, we noticed that the increase in glutathione, IL-6, and IL-8 was somewhat less compared to the first exposure cycle in cell cultures exposed to DE-AGGR and SuperN suspensions. It appears that the cells stressed during first exposure cycle, undergoing recovery probably due to protective effects induced during the first exposure cycle. Moreover, cell viability at the end of both exposure cycles remained similar to control. Further research is needed to verify whether the decreased cell growth was the result of cell cycle arrest and/or cell death (apoptosis). Nevertheless, the cells recovered similarly to the control one week after exposure was discontinued, indicating that the response observed was due to continuous exposure to SAS. This finding is particularly important as this indicates that continuous human exposure to SAS results in elevated levels of biological responses, which could lead to adverse effects.

Numerous studies have reported that short-term in vivo exposure to SAS elevated the levels of LDH, IL-6, IL-8, and GSH depletion in the lung [15]. However, long-term and repeated exposure in vivo studies for SAS are scarce. In a study [34], rats were exposed to 50 mg/m^3^ of SAS for 6 h/day, 5 days/week for 13 weeks and effects were characterized after 6.5 weeks and 13 weeks of exposure, and after three and eight months of recovery. An increase in cytotoxicity biomarkers (LDH) and inflammatory cells was noticed after 6.5 and 13 weeks, but the effects were significantly mitigated after both recovery periods. Genotoxicity was not observed at any of these time points. In another study [35], rats were exposed to 50 mg/m^3^ of SAS for 6 h/day for five days and adverse effects were characterized after last exposure or one or three months later. SAS induced elevated levels of cytotoxicity biomarkers and lung damage after last exposure, but the effects were reversed three months post exposure. In our study, we observed that the effects induced by DE-AGGR and PREC were reversed after a one week recovery period. This suggests that our long-term exposure design may be appropriate to predict the in vivo adverse outcome of repeated exposure to NMs.

## 5. Conclusions

In this study, we demonstrated the toxicological relevance of AA in a repeated low dose in vitro exposure study. Neither TiO_2_ exposure nor their agglomeration state affected the measured biological endpoints, possibly due to insufficient applied dose. On the other hand, we noticed that a fraction of SAS aggregates in their manufactured form (2.5 µm) did not appear biologically less active compared to nano-sized SAS produced by sonication. Apparently, in vitro studies with more biological endpoints and animal studies are required to verify these results. Moreover, further characterization is needed to reveal properties other than size that make SuperN fractions biologically more active. Since SAS used in this study is a representative of SAS approved as a food additive (E551), more attention needs to be paid in the future to the possible adverse effects of SuperN fractions, particularly their long-term effects. The results of this study also might spur toxicologists to perform more long-term studies in the future to reveal the toxicological relevance of other NMs that are agglomerated/aggregated in their manufactured form.

## Data Availability

The data presented in this study are available on request from the corresponding authors.

## Supplementary Materials

The following are available online at https://www.mdpi.com/article/10.3390/nano11071793/s1: Figure S1: Representative TEM micrographs of freshly prepared TiO_2_ stock suspensions, Figure S2: Representative bright field microscopic images of freshly prepared SAS stock suspensions, Figure S3: Influence of SAS aggregation on cytotoxicity and biological responses, Table S1: Size characterization of freshly prepared TiO_2_ stock suspensions, Table S2: Size characterization of freshly prepared TiO_2_ stock suspensions, Table S3: Characterization of freshly prepared SAS stock suspensions, Table S4: Z-average sizes (measured by DLS) of SAS suspensions in different cell culture medium (100 μg/mL).
