# Supplementary material for: Assessing the Toxicological Relevance of Nanomaterial Agglomerates and Aggregates Using Realistic Exposure In Vitro
Source: Nanomaterials (Basel). 2021 Jul 9;11(7):1793. doi: 10.3390/nano11071793 (PMC8308261; doi:10.3390/nano11071793)
Supplement: Supplementary file 1 [file nanomaterials-11-01793-s001.zip › nanomaterials-1285498-supplementary.pdf]

Supplementary file

Assessing the Toxicological Relevance of Nanomaterial Agglomerates and Aggregates Using Realistic Exposure In Vitro

Sivakumar Murugadoss [1], Lode Godderis [2,3], Manosij Ghosh [1] and Peter H. Hoet [1,*]

[1] Laboratory of Toxicology, Unit of Environment and Health, Department of Public Health and Primary Care, KU Leuven, 3000 Leuven, Belgium; sivakumar.murugadoss@kuleuven.be (S.M.); manosij.ghosh@kuleuven.be (M.G.)
[2] Laboratory for Occupational and Environmental Hygiene, Unit of Environment and Health, Department of Public Health and Primary Care, KU Leuven, 3000 Leuven, Belgium; lode.godderis@kuleuven.be
[3] IDEWE, External Service for Prevention and Protection at work, Interleuvenlaan 58, 3001 Heverlee, Belgium
* Correspondence: peter.hoet@kuleuven.be; Tel.: +32-1633-0197

Figure S1 and Table S1—provided in supplementary as they are published elsewhere [1].

Figure S2 and Table S3—provided in supplementary as they are published elsewhere [2].

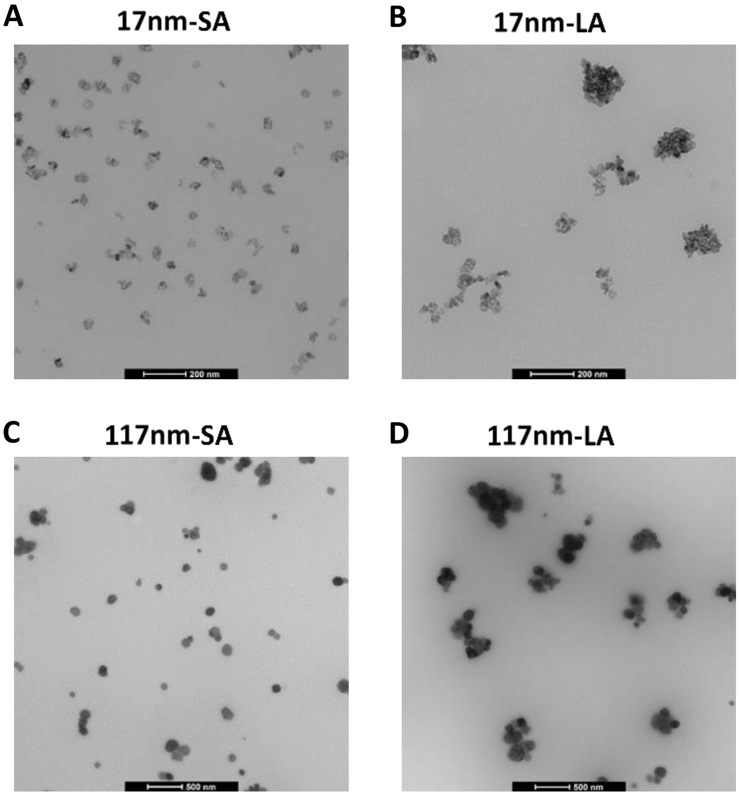

Figure S1. Representative TEM micrographs of freshly prepared TiO_2 stock suspensions of small (SA) and large agglomerates (LA). 17nm-SA (**A**), 17nm-LA (**B**), 117nm-SA (**C**) and 117nm-LA (**D**).

Table S1. Characterization of freshly prepared TiO_2 stock suspensions (2.56 mg/mL).

Stock Suspensions	Description	TEM			PTA	DLS	DLS
		Median ECD (nm)	Mean ECD (nm)	Mean Feret min (nm)	Mean Hydrodynamic size (nm)	Z-average (nm)	Zeta potential (mV)
17nm-SA	Small agglomerates of 17 nm sized TiO_2 NP	18	100	33	134	600	33
17nm-LA	Large agglomerates of 17 nm sized TiO_2 NP	127	200	120	207	900	−37
117nm-SA	Large agglomerates of 117 nm sized TiO_2 NP	122	250	148	259	280	−46
117nm-LA	Large agglomerates of 117 nm sized TiO_2 NP	352	500	309	221	580	15

Median and mean equivalent circle diameter (ECD) and mean feret minimum (feret min) measured by transmission electron microscopy (TEM), Z-average (mean hydrodynamic size) by dynamic light scattering (DLS) and mean hydrodynamic size by particle tracking analysis (PTA).

Table S2. Z-average sizes (measured by DLS) of TiO_2 suspensions in different cell culture medium (100 µg/mL) at different time points.

CCM	17nm-SA		17nm-LA		117nm-SA		117nm-LA	
	0h	24h	0h	24h	0h	24h	0h	24h
HBE	685.2	575.3	784	779.8	338.9	305.6	574.5	788
Caco2	583.8	463.8	753.9	705.6	349.5	353.5	615.7	706.2
THP-1	575.9	594.8	886	907	348.8	343.8	569	635.1

CCM—complete culture medium.

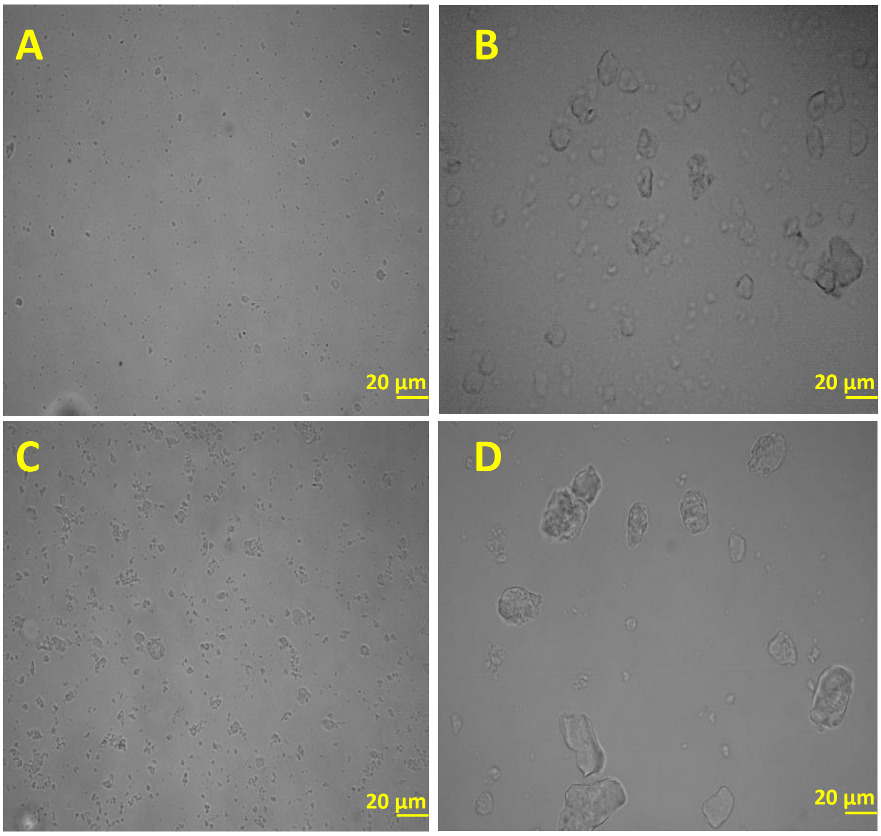

Figure S2. Representative bright field microscopic images of freshly prepared SAS stock suspensions. DE-AGGR (**A**), AGGR (**B**), SuperN (**C**) and PREC (**D**). Scale bar -20µm.

Table S3. Characterization of freshly prepared SAS stock suspensions.

**Stock Suspensions**	**Description**	**Stock Concentration (mg/mL)**	TEM		DLS	BF	DLS
			Mean ECD (nm)	Mean Feret min (nm)	Z-average (nm)	Approx. average diameter (µm)	Zeta potential (mV)
DE-AGGR	De-aggregated suspension	2.56	100	28	264	n/a	−33
AGGR	Aggregated suspension	2.56	2000	600	1,2530	n/a	n/a
SuperN	Non-precipitating fraction of AGGR	0.64	600[a]	n/a	3953	2.5	n/a
PREC	Precipitating fraction of AGGR	1.92	750[a]	n/a	3332	25	n/a

Median and mean equivalent circle diameter (ECD) and mean feret minimum (feret min) measured by transmission electron microscopy (TEM), Z-average (mean hydrodynamic size) by dynamic light scattering (DLS) and average diameter by bright field microscopy (BF). n/a - not available due to their quick sedimentation while performing zeta potential measurements.

Table S4. Z-average sizes (measured by DLS) of SAS suspensions in different cell culture medium (100 μg/mL).

CCM	DE-AGGR		AGGR		SuperN		PREC	
	0h	24h	0h	24h	0h	24h	0h	24h
HBE	220.4	207.7	2087	2353	4507	3851	1101	957
Caco2	155.2	178.9	1049	151.7	4500	2233	1370	1376
THP-1	162.2	166.4	3359	3057	5507	3166	1571	955

CCM—complete culture medium.

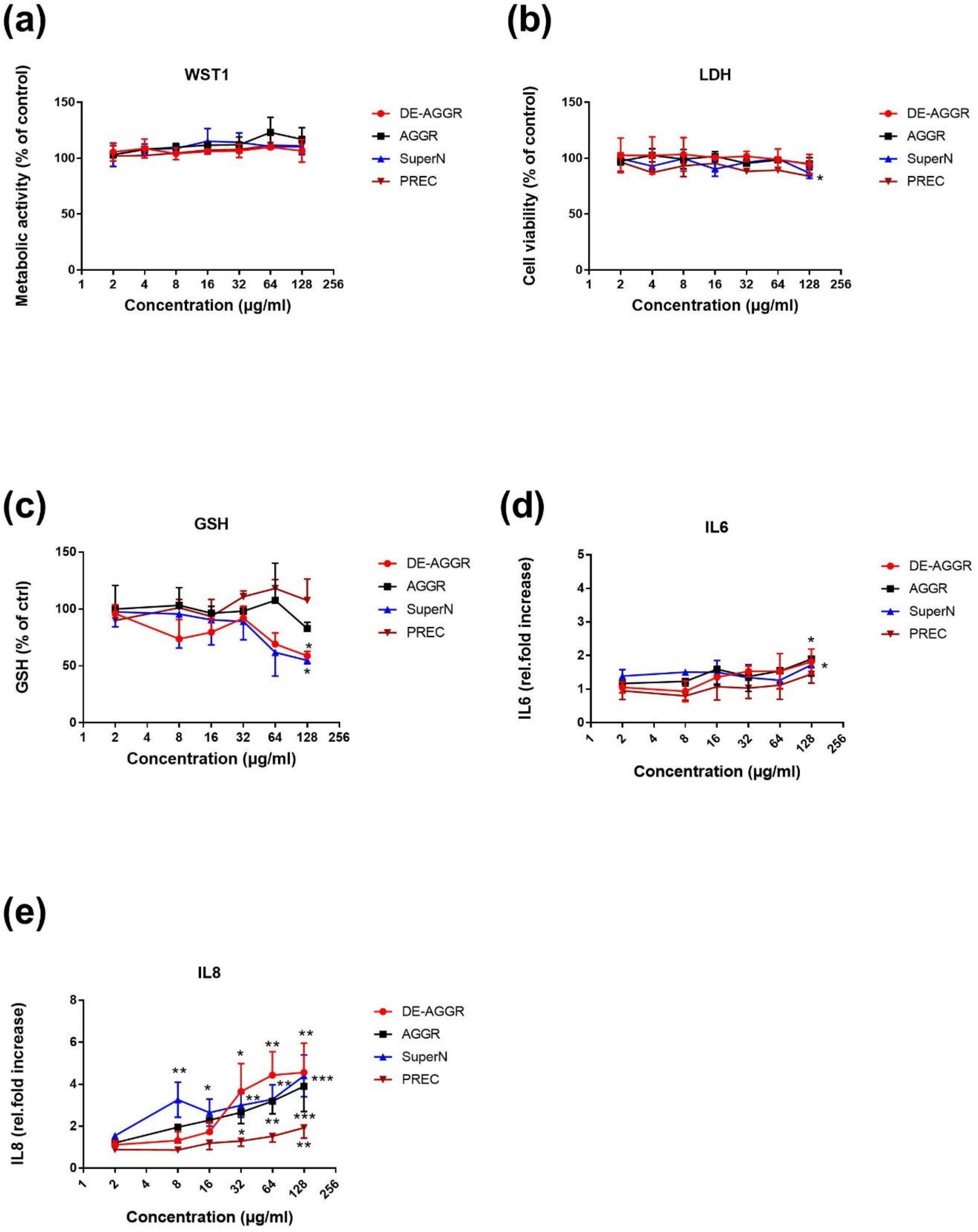

Figure S3. Influence of SAS aggregation on cytotoxicity and biological responses. Effect on cell metabolic activity (**a**), cell viability (**b**), total glutathione (**c**), IL-6 (**d**) and IL-8 secretion (**e**) measured in HBE after 24h exposure to different SAS suspensions. The exposures were performed in the presence of serum. Methods to measure different endpoints were provided in detail in [2]. Data are expressed as means ± SD from three independent experiments performed in triplicates or duplicates. $p < 0.05$ (*), $p < 0.01$ (**) and $p < 0.001$ (***) represent significant differences compared to control (One-way ANOVA followed by Dunnett's multiple comparison test).

References

1. Murugadoss, S.; Brassinne, F.; Sebaihi, N.; Petry, J.; Cokic, S.M.; Van Landuyt, K.L.; Godderis, L.; Mast, J.; Lison, D.; Hoet, P.H.; et al. Agglomeration of titanium dioxide nanoparticles increases toxicological responses in vitro and in vivo. *Part. Fibre Toxicol.* **2020**, *17*, 1–14.
2. Murugadoss, S.; Brule, S.V.D.; Brassinne, F.; Sebaihi, N.; Mejia, J.; Lucas, S.; Petry, J.; Godderis, L.; Mast, J.; Lison, D.; et al. Is aggregated synthetic amorphous silica toxicologically relevant? *Part. Fibre Toxicol.* **2020**, *17*, 1–12.
